# Influence of Baoyuan decoction and Taohong Siwu decoction combined with Western medicine on serum lipid levels in elderly patients with coronary heart disease

**DOI:** 10.5937/jomb0-52017

**Published:** 2025-08-21

**Authors:** Bingrui Wu, Shenggao Zhang, Ruihong Zhou

**Affiliations:** 1 Suzhou City Integrated Traditional Chinese and Western Medicine Hospital, Heart Disease Department, Suzhou, China

**Keywords:** Baoyuan and Taohong Siwu decoctions, western medicine, coronary heart disease in elderly patients, serum indicators, Baoyuan i Taohong Siwu dekokti, zapadna medicina, koronarna bolest srca kod starijih pacijenata, serumski pokazatelji

## Abstract

**Background:**

To explore how Baoyuan and Taohong Siwu decoctions combined with Western medicine affect triglycerides (TG), total cholesterol (TC), low-density lipoprotein cholesterol (LDL-C), and high-density lipoprotein cholesterol (HDL-C) in elderly patients with coronary heart disease.

**Methods:**

Ninety elderly patients with coronary heart disease who were hospitalized from January 2021 to December 2022 were selected and divided into two groups based on different treatment methods, with 45 patients in each group. The control group was treated with Western medicine alone, while the research group was treated with the combination of Baoyuan and Taohong Siwu decoctions based on the control group. The improvement of traditional Chinese medicine symptom scores, various blood lipid levels, and serum indicators of cardiac function were compared between two groups before treatment, 1 month, 2 months, and 3 months of treatment.

**Results:**

Before treatment, the two groups had identical Traditional Chinese Medicine symptom scores, various blood lipid levels, and various serum indicators of cardiac function (P> 0.05). After treating 1 month, 2 months, and 3 months, the chest pain, tightness, fatigue, and sweating scores in the research group were lower (P< 0.05). The TG, TC, LDL-C, and HDL-C levels in the research group were lower (P< 0.05). The levels of plasma N-terminal brain natriuretic peptide (NT-proBNP) and monocyte chemoattractant protein-1 (MCP-1) in the research group were lower (P <0.05).

**Conclusions:**

Combining Baoyuan and Taohong Siwu decoctions and Western medicine treatment can help improve the symptoms and signs of coronary heart disease in the elderly. It can reduce the levels of various indicators such as LDL-C, TG, TC, and HDL-C in the body and promote the improvement of cardiac function.

## Abstract (sr)

**Uvod:**

Cilj studije je bio da se istraži kako kombinacija Baoyuan i Taohong Siwu dekokta sa zapadnom medicinom utiče na trigliceride (TG), ukupan holesterol (TC), holesterol niskog denziteta lipoproteina (LDL-C) i holesterol visokog denziteta lipoproteina (HDL-C) kod starijih pacijenata sa koronarnom bolešću srca.

**Metode:**

Izabrano je 90 starijih pacijenata sa koronarnom bolešću srca koji su bili hospitalizovani od januara 2021. do decembra 2022. godine i podeljeni su u dve grupe na osnovu različitih metoda lečenja, sa po 45 pacijenata u svakoj grupi. Kontrolna grupa je lečena isključivo zapadnom medicinom, dok je grupa nad kojom je sprovedeno istraživanje lečena kombinacijom Baoyuan i Taohong Siwu dekokta na osnovu lečenja kontrolne grupe. Upoređeno je poboljšanje simptoma po skoru tradicionalne kineske medicine, različitih nivoa krvnih lipida i serumskih pokazatelja srčane funkcije između dve grupe pre lečenja, mesec dana, dva meseca i tri meseca nakon lečenja.

**Rezultati:**

Pre lečenja, obe grupe su imale iste rezultate u pogledu simptoma po skorovima tradicionalne kineske medicine, različite nivoe krvnih lipida i različite serumske pokazatelje srcane funkcije (P > 0,05). Nakon mesec dana, dva meseca i tri meseca lečenja, rezultati za bolove u grudima, stezanje u grudima, umor i znojenje u grupi nad kojom je sprovedeno istraživanje su bili niži (P< 0,05). Nivoi TG, TC, LDL-C i HDL-C u toj grupi su bili niži (P < 0,05). Nivoi plazmatskog NT-proBNP (N-terminalan natriuretski peptid mozga) i MCP-1 (monocitni hemotaktički protein-1) u grupi na kojoj je vršeno istraživanje su bili niži (P< 0,05).

**Zaključak:**

Kombinacija Baoyuan i Taohong Siwu dekokta i zapadne medicine može da pomogne u poboljšanju simptoma i znakova kod starijih pacijenata sa koronarnom bolešću srca. Ona može da smanji nivoe različitih pokazatelja kao što su LDL-C, TG, TC i HDL-C u organizmu, i da podstakne poboljšanje srčane funkcije.

## Introduction

Coronary heart disease (CHD) is a disease with high incidence in the cardiology department. It has a high incidence rate among middle-aged and elderly people. Data from an epidemiological survey shows that [1] the incidence rate of global diseases can reach 32%, while the incidence rate of CHD among the overall incidence population in China can reach 36.0%, affecting and threatening individual health and life. The abnormal blood lipid level is the main reason for the progress of CHD. With the gradual increase of blood lipid level, coronary atherosclerotic plaque gradually forms, significantly the abnormal increase of high-density lipoprotein cholesterol level in blood lipid indicators, which will significantly increase the mortality of the disease [2].

At present, the treatment of chronic CHD in the elderly mainly relies on drugs and assists in daily life regulation to maintain a stable state. The main clinical medications include anti-platelet aggregation, lipid-lowering, and anticoagulant therapy. The main drugs selected include nitrates, P-receptor blockers, platelet inhibitors, etc. Through comprehensive treatment with drugs, it can improve myocardial ischemia, reduce myocardial oxygen consumption, and ultimately lead to symptoms of angina pectoris [3]. Metoprolol is one of the most commonly used drugs in Western medicine treatment. It is a β-receptor blocker with high selectivity. It can reduce myocardial autonomy, promote the rise of ventricular fibrillation threshold, alleviate conduction nerve velocity, avoid and prevent ventricular bradycardia, and reduce the incidence rate of ventricular fibrillation [4]. However, the clinical study [5] has shown that although the use of metoprolol alone can improve the condition, the drug may have specific adverse reactions, mainly manifested as bradycardia, chest tightness, and fatigue. This not only affects treatment but also affects the condition and reduces treatment effectiveness. Therefore, when treating the elderly with CHD, effective and safe treatment measures are crucial for the recovery of the condition.

Baoyuan Decoction and Taohong Siwu Decoction (BYD-THSWD) is a group of traditional formulas that have the effects of resolving phlegm, dispersing nodules, promoting qi and promoting blood circulation [6]. Traditional Chinese medicine is gradually being applied in treating coronary heart disease, which can improve the treatment effect to a certain extent by evaluating the overall state of individuals by combining dialectical medication with Western medicine treatment. Previous studies have shown that the combination of Baoyuan Decoction and Taohong Siwu Decoction can achieve significant therapeutic effects and promote the prognosis of coronary heart disease patients. However, there have been few studies in the past on combining Baoyuan Decoction and Taohong Siwu Decoction with Western medicine in treating coronary heart disease. The novelty of the article lies in the combined application of Baoyuan Decoction, Taohong Siwu Decoction, and Western medicine in the treatment of elderly patients with coronary heart disease and the analysis of the disease treatment effect. Therefore, in this study, the combination of BYD-THSWD and Western medicine was used to treat elderly CHD patients and the therapeutic effect of the disease was analyzed. The following is the report.

## Materials and methods

### Research materials

Ninety elderly CHD patients treated from January 2021 to December 2022 were regarded as research subjects. They were separated into two groups by different treatments, with 45 people in each group. The two groups had the same gender, age, body mass index (BMI), height, course of disease, comorbidities, and CHD grading (*P*>0.05) in Table 1.

**Table 1:** Comparison of Baseline Data (x̄±s, %).

Group	n	Sex		Age<br>(year old)	BMI<br>(kg/m^2^)	Height<br>(m)	Disease course<br>(year)
		Male	Female				
CG	45	26	19	62.49±1.27	23.54±2.06	1.67±0.15	3.56±1.16
RG	45	23	22	62.71 ±1.36	23.91 ±1.86	1.70±0.11	3.81 ±1.02
*t*		0.403		0.793	0.894	1.082	1.086
*P*		0.525		0.430	0.374	0.282	0.281
Group	n	Merged underlying diseases			CHD grade		
		Hypertension	Hyperlipidemia		Level I	Level II	Level III
CG	45	12	15	18	27	13	5
RG	45	14	11	20	23	15	7
*t*		0.875			0.796		
*P*		0.646			0.672		

### Inclusion and exclusion criteria

Inclusion criteria: (A) Confirmed CHD, meeting the criteria in the literature [7]. (B) Medication treatment was implemented in the hospital for the first time. (C) The type of TCM syndrome differentiation disease was Qi deficiency and blood stasis type. (D) More than 2 episodes of angina per week. (E) The cardiac function grading was between I and III.

Exclusion criteria: (A) History of concomitant myocardial infarction and unstable angina. (B) Cardiac function grading was above level IV. (C) Long-term drug abuse and alcohol abuse. (D) The combination was called severe diabetes and hypertension, and the condition couldn't be controlled. (E) Complicated with severe hemorrhagic diseases, severe cerebrovascular diseases, and malignant tumours. (F) Mental disorders, cognitive dysfunction.

### Research methods

Control group (CG): Simple implementation of Western medicine treatment, including anti-platelet therapy, lipid-lowering therapy, and vasodilator therapy. Aspirin 50-100 mg/day, once per day. Rosuvastatin 10-20 mg/day, taken orally at night, once a day. Metoprolol 25-50 mg/dose orally, 2 times per day. One month of continuous treatment and three months of continuous treatment were a course.

Research group (RG): Combined with BYD-THSWD treatment based on CG. Western medicine has the same dosage as CG, and BYD-THSWD is selected for treatment. TCM formula: 15 g peach kernel, 15 g red peony, 20 g Astragalus membranaceus, 15 g cassia twig, 15 g Codonopsis pilosula, 9 g Ligusticum wallichii, 20 g Ophiopogon japonicus, 15 g Caulis spatholobi, 10 g Angelica sinensis, 6 g Radix liquiritiae, 10 g Safflower, 15 g fried semen ziziphi spinosae. Before medication, the patient should undergo syndrome differentiation, and the medication formula should be adjusted according to the symptoms. For those with severe congestion, 9 g curcuma zedoary, 4 g leech, and 9 g trigone should be added. Add 15 g yam and 15 g fried Atractylodes macrocephala Koidz for those with severe fatigue. For insomnia, add 15 g tube flue flower stem. For severe chest tightness, add 15 g Fructus Aurantii. The medication is decocted in water, 1 dose per day, with a treatment of 1 month and a continuous treatment of 3 months.

### Observation indicators and evaluation

TCM Symptom Scoring: Based on the reference [7,8], there are four main evaluation items, including chest pain, fatigue, chest tightness, and sweating. Symptoms range from mild to severe, with 0-5 points. The higher the score, the more severe the disease symptoms are.

Various blood lipid levels: The examination items include triglyceride (TG), total cholesterol (TC), low-density lipoprotein cholesterol (LDL-C), and high-density lipoprotein cholesterol (HDL-C). 5 mL of fasting blood from the patient was centrifuged at 5500 r/min for 1 minute to obtain the supernatant. The detection was conducted using a fully automated blood analysis instrument.

Serum indicators of cardiac function: The examination indicators include plasma N-terminal brain natriuretic peptide (NT-proBNP) and Monocyte chemotactic protein 1 (MCP-1). 5mL of venous blood was taken from the patient on an empty stomach. NT-proBNP was analyzed using electrochemiluminescence immunoassay, while MCP-1 was examined using enzyme-linked immunosorbent assay.

### Statistical analysis

The data were all processed by SPSS25.0, and the counting data are displayed in terms of number of cases (n) and rate (%). χ^2^ tests are conducted between groups. The econometric data that conform to the Chint distribution are described as mean ± standard deviation (x̄±s), and independent sample t-tests are performed between groups.* P*<0.05 indicates statistical significance between data.

## Results

### Comparison of baseline data

In Table 1, two groups had the same gender, age, BMI, height, course of disease, comorbidities, and CHD grading (*P*>0.05).

### TCM Symptom Scores

Before treatment, both groups had the same TCM symptom score (*P*>0.05). After being treated at different times, the scores of chest pain, chest tightness, fatigue, and sweating decreased in both groups, with RG lower than CG (*P*<0.05) in Table 2.

**Table 2:** TCM Symptom Scores for Two Groups (x̄±s, point). Note: ^*^Compared with before treating, P<0.05. ^*#^Compared with the 1-month treatment, P<0.05. ^*#&^Compared with the 2-month treatment, P<0.05.

Group	n	Chest pain				Chest tightness			
		Before	1 month<br>after	2 months<br>after	3 months<br>after	Before	1 month<br>after	2 months<br>after	3 months<br>after
CG	45	2.48±0.48	2.01 ±0.54^*^	1.87±0.30^*#^	1.69±0.19^*#&^	2.51 ±0.38	1.99±0.25^*^	1.81±0.17^*#^	1.65±0.14^*#&^
RG	45	2.45±0.51	1.85±0.29^*^	1.68±0.22^*#^	1.31 ±0.12^*#&^	2.61 ±0.27	1.80±0.21^*^	1.73±0.15^*#^	1.43±0.10^*#&^
*t*		0.287	2.402	3.426	11.343	1.439	3.904	2.367	8.578
*P*		0.775	0.018	0.001	0.001	0.154	0.002	0.020	0.000
	n	Feeble				Sweating			
		Before	1 month<br>after	2 months<br>after	3 months<br>after	Before	1 month<br>after	2 months<br>after	3 months<br>after
CG	45	2.71 ±0.26	1.98±0.23^*^	1.76±0.15^*#^	1.51 ±0.11^*#&^	2.44±0.71	1.93±0.54^*^	1.78±0.43^*#^	1.34±0.34^*#&^
RG	45	2.45±0.29	1.80±0.16^*^	1.55±0.12^*#^	1.30±0.03^*#&^	2.38±0.65	1.71 ±0.41^*^	1.41 ±0.29^*#^	1.02±0.19^*#&^
*t*		4.478	4.310	7.334	12.355	0.418	2.177	4.786	5.511
*P*		0.000	0.000	0.000	0.000	0.677	0.032	0.000	0.000

### Comparison of TG, TC, LDL-C, and HDL-C

Before treatment, both groups had the same levels of various blood lipid indicators (*P*>0.05). After treating different times, both groups' TG, TC, LDL-C, and HDL-C decreased, while RG was lower than CG (*P*<0.05) in Table 3.

**Table 3:** TG, TC, LDL-C, and HDL-C (x̄±s, mmol/L).

Group	n	TG				TC			
		Before	1 month <br>after	2 months<br>after	3 months<br>after	Before	1 month<br>after	2 months<br>after	3 months<br>after
CG	45	6.82±2.13	5.42±1.78^*^	4.00±1.39^*#^	2.97±1.10^*#&^	6.49±2.08	5.11 ±1.85^*^	4.13±1.57^*#^	3.10±0.72^*#&^
RG	45	6.50±2.20	4.06±1.56^*^	3.06±1.18^*#^	1.26±0.67^*#&^	6.63±2.17	4.20±1.69^*^	3.24±1.31^*#^	2.19±0.39^*#&^
*t*		0.701	3.855	3.458	8.906	0.312	2.436	2.920	7.455
*P*		0.485	0.000	0.001	0.000	0.755	0.017	0.004	0.000
	n	LDL-C				HDL-C			
		Before	1 month <br>after	2 months<br>after	3 months after	Before	1 month<br>after	2 months<br>after	3 months<br>after
CG	45	7.63±1.99	6.00±1.57^*^	5.97±1.45^*#^	4.18±1.00^*#&^	6.15±1.01	5.16±0.87^*^	4.76±0.51^*#^	3.00±0.26^*#&^
RG	45	7.71 ±1.78	5.01 ±1.31^*^	4.59±1.13^*#^	3.10±0.51^*#&^	6.26±1.07	4.79±0.75^*^	3.53±0.31^*#^	1.23±0.04^*#&^
*t*		0.201	3.248	5.036	6.454	0.501	2.161	13.825	45.136
*P*		0.841	0.002	0.000	0.000	0.617	0.033	0.000	0.000

**Figure 1:**
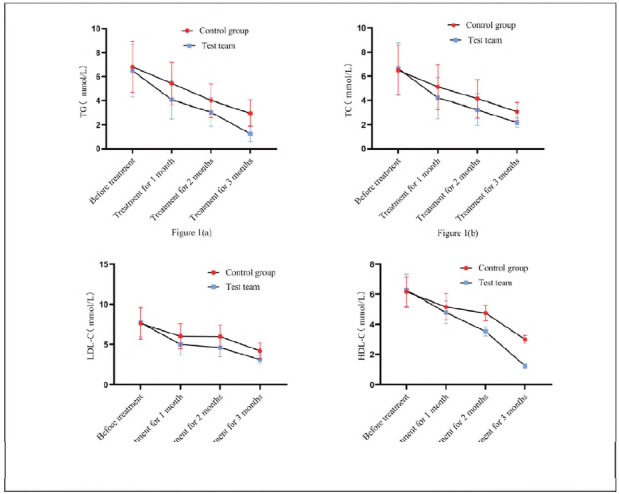
Comparison of TG, TC, LDL-C, and HDL-C.<br>Note: Figure 1 (a) shows TG, Figure 1 (b) shows TC, Figure 1 (c) shows LDL-C, and Figure 1 (d) shows HDL-C. The red circular curve represents CG. The blue square curve represents RG. The curve fluctuation indicates the changes in various blood lipid indicators before and after treatment in both groups.

Compared with CG treated with Western medicine alone, RG with BYD-THSWD showed decreased TG, TC, LDL-C, and HDL-C levels. Figure 1 (a), 1 (b), 1 (c), and 1 (d) showed the trend changes of various indicators. Two groups had the same TG, TC, LDL-C, and HDL-C levels before treatment (*P*>0.05) and were treated for 1 and 2 months. After 3 months, the TG, TC, LDL-C, and HDL-C levels in RG were lower than CG's (*P*<0.05).

### Comparison of serum factors NT-proBNP and MCP-1

Before treatment, both groups had the same cardiac function indicators (*P*>0.05). After treatment for 1 month, 2 months, and 3 months, NT-proBNP and MCP-1 decreased in both groups, and RG was lower than CG (*P*<0.05) in Table 4.

**Table 4:** Comparison of NT-proBNP and MCP-1 (x̄±s). Note: "Compared with before treating, P<0.05. ^*#^Compared with the 1-month treatment, P<0.05. ^*#&^Compared with the 2-month treatment, P<0.05.

Group	n	MCP-1 (pg/mL)				NT-proBNP (ng/mL)			
		Before	1 month<br>after	2 months<br>after	3 months<br>after	Before	1 month<br>after	2 months<br>after	3 months<br>after
CG	45	156.54±<br>12.74	141.06±<br>11.06^*^	132.18±<br>8.37^*#^	126.38±<br>7.04^*#&^	1024.52±<br>123.09	916.34±<br>110.34^*^	816.39±<br>84.37^*#^	781.28±<br>68.29^*#&^
RG	45	156.19±<br>12.57	135.29±<br>10.09^*^	127.34±<br>7.16^*#^	113.09±<br>5.49^*#&^	1024.37±<br>123.13	804.28±<br>98.74^*^	700.25±<br>71.04^*#^	614.28±<br>54.77^*#&^
t		0.057	2.585	2.948	3.000	0.006	5.077	7.064	12.797
P		0.955	0.011	0.004	0.004	0.995	0.000	0.000	0.000

**Figure 2:**
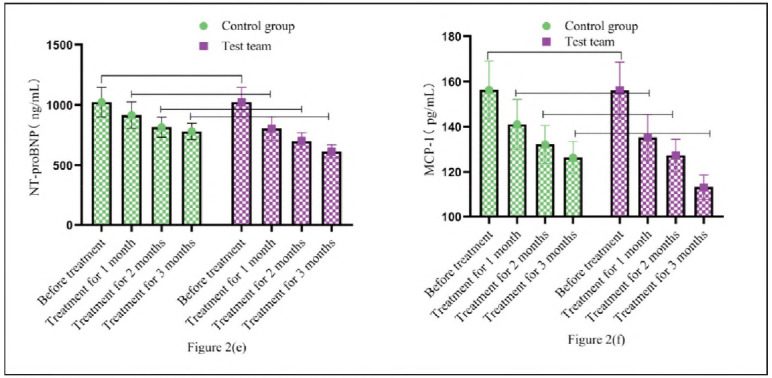
Comparison of serum factors NT-proBNP and MCP-1.<br>Note: Figure 2 (e) shows NT-proBNR and Figure 2 (f) shows MCP-1. The green circular bar chart is CG. The purple square bar chart shows RG. The high and low levels of the bar chart represent the changes in various serum indicators before and after treatment in both groups.

Compared with CG treated with Western medicine alone, RG combined with BYD-THSWD showed a decrease in NT-proBNP and MCP-1. Figure 2 (e) and 2 (f) show the trend changes in the levels of various indicators. Before treatment, the two groups had the same levels of NT-proBNP and MCP-1 (*P*>0.05). After treatment at different times, NT-proBNP and MCP-1 in RG were lower than CG's (*P*<0.05) (Figure 2).

## Discussion

### The significance of elderly CFID combined with BYD-TFISWD and Western medicine treatment

Modern medical research has found that [8] [9] the pathogenesis of CHD is closely related to coronary atherosclerosis. When plaque is unstable, it will increase the formation of intravascular thrombosis. This ultimately leads to a state of microcirculation in the myocardium, leading to microvascular blockage and insufficient myocardial supply, leading to the occurrence of diseases. The research of Jurisch D et al. [10] shows that coronary atherosclerosis is an important pathological basis for CHD, and the body's inflammatory response is also closely related to coronary atherosclerosis. The Western medicine metoprolol has the effects of lowering blood pressure and regulating heart rate, which can control the clinical symptoms of CHD angina patients. However, most elderly patients have poor physical fitness, more comorbidities with underlying diseases, and a high incidence of adverse reactions during medication, resulting in the inability to achieve ideal therapeutic effects solely with medication. According to TCM theory [11], the occurrence of CHD can be classified into the category of chest obstruction. The occurrence of blood stasis and qi deficiency in the body is an important pathogenesis of the disease, and the heart is the main location of the disease. It belongs to the syndrome of deficiency and excess, with stasis of blood blocking the meridians as the criterion and deficiency of visceral qi as the basis. The occurrence factors of elderly CHD are mainly due to various factors such as old age, weak physique, insufficient endowment, poor mood, improper diet, and internal injuries caused by labour and illness. They lead to heart qi depletion, inability to pass blood, and ultimately result in myocardial blood vessel stasis, poor blood flow, and chest pain. This study selected BYD-THSWD treatment with ingredients including Radix liquiritiae, Astragalus membranaceus, Ginseng, Cinnamomum cassia. The drug can tonify qi and warm yang and has a significant therapeutic effect on symptoms of qi deficiency and blood stasis combined with yang deficiency [12]. Liang X et al. [13] showed that BYD-THSWD could protect the myocardium through multi-component and multi-target network controlled regulation, reduce the occurrence of myocardial ischemia and hypoxia, and avoid adverse reactions during treatment. Based on the above analysis and research, the combination of Baoyuan therapy is the key to improving the treatment effect of elderly CHD. Therefore, the combination of BYD-THSWD in treating elderly CHD is of great significance.

### The effect of BYD-THSWD combined with Western medicine on the symptom score of TCM in elderly patients with CHD

The results of this study showed that RG scores of various TCM symptoms in the combined implementation of BYD-THSWD were lower than CG, consistent with the research results of Ji Z et al. [14]. The main reasons include that the BYD-THSWD formula mainly focuses on removing blood stasis as the treatment core, and is supplemented by promoting qi and nourishing blood. The Peach kernel in the formula is a powerful blood-breaking product. Peach kernel and Safflower can promote blood circulation and remove blood stasis. Angelica sinensis can nourish blood, regulate menstruation, and nourish yin and liver. Red peonies can nourish blood and assist in replenishing qi. Ligusticum wallichii can promote blood and Qi circulation, regulate Qi and blood flow, and achieve effective blood circulation. The proper combination of all prescription drugs can eliminate congestion, generate new blood, achieve smooth Qi circulation, and improve various clinical symptoms in TCM [15].

### The effect of BYD-THSWD combined with Western medicine on blood lipid levels in elderly CHD patients

TG, TC, LDL-C, HDL-C, etc., are important lipid indicators, and their elevated levels can predict cardiovascular disease caused by atherosclerosis. Especially in the risk prediction of CHD diseases, they have important value. This study confirmed that compared with CG treated with Western medicine alone, RG treated with BYD-THSWD combined with BYD-THSWD showed a decrease in various blood lipid levels. Zhang X et al. [16] showed that BYD-THSWD effectively reduced blood lipid levels and controlled disease status in CHD angina patients, consistent with this study. The main drugs in the BYD-THSWD drug formula include Codonopsis pilosula and Astragalus membranaceus, which replenish qi and blood. Angelica sinensis can nourish and promote blood circulation. Peach kernel, Safflower, Red peony, Ligusticum wallichii can promote blood circulation and remove blood stasis. Cinnamon can promote blood circulation and relieve pain, while Radix liquiritiae can slow and relieve pain. Many prescriptions can play a role in tonifying qi, promoting blood circulation, unblocking collaterals, and relieving pain by adding or subtracting medication after syndrome differentiation. Zheng et al. [16], reported that Astragalus membranaceus can improve blood viscosity by positively stimulating the heart through its main component Astragalus membranaceus saponins [17]. The main components in Codonopsis pilosula include Codonopsis pilosula glycosides, Codonopsis pilosula polysaccharides, etc., which can protect the myocardium and effectively inhibit the formation of blood clots, α-pinene, β-pinene, and cinnamon components in Angelica sinensis and Cassia twig can effectively promote coronary artery dilation, increase blood flow, and improve myocardial ischemia. The main components in Peach kernel, Safflower, Red peony, Ligusticum wallichi include amygdalin, Safflower saponin, paeoniflorin, Ligusticum wallichi zine, etc., which can dilate myocardial blood vessels and inhibit platelet aggregation. The combined application of many drugs can promote the improvement of myocardial ischemia symptoms and alleviate the damage caused by ischemia and hypoxia to the myocardium. Combined medication can effectively control the body's inflammatory response and protect the myocardium, thereby reducing the levels of various blood lipid indicators.

### The effect of BYD-THSWD combined with Western medicine on NT-proBNP and MCP-1 in elderly patients with CHD

NT-proBNP is a commonly used indicator in clinical cardiac function assessment. It can effectively evaluate the cardiac function status and provide a basis for clinical disease treatment based on its level changes, combined with the patient's clinical symptoms and signs. However, this indicator level change can also increase in the blood of patients with renal insufficiency or infection. Therefore, when applying this indicator to evaluate cardiac function, a comprehensive evaluation of clinical symptoms is needed [18] [19]. MCP-1 is mainly involved in the whole process of migration, adhesion and foam of monocyte macrophages in the body. It can promote the production of proinflammatory cytokines, produce inflammatory mediators, and aggravate inflammatory reactions. Maldonado Ruiz R et al. [20] showed that MCP-1 can be an important indicator for evaluating the inflammatory response process during the onset of CHD angina. This study confirms that RG application of BYD-THSWD can reduce NT-proBNP and MCP-1. It may be due to the combination of BYD-THSWD treatment based on the pharmacological effects of Western medicine, which exerts the greatest advantage in treatment. It can balance the imbalance of yin and yang in the organs of elderly CHD and pay attention to the prognosis of disease symptoms and blood lipid levels. The maximum effect of drug application can be exerted through syndrome differentiation and treatment. Promoting the improvement of symptoms and blood lipids can reduce the level of cardiac function indicators [21].

### Strengths and limitations

The advantage of the article lies in the combination of Baoyuan Decoction and Taohong Siwu Decoction with modified treatment based on the pharmacological effects of Western medicine. Its greatest advantage is to balance the imbalance of visceral yin and yang in elderly patients with coronary heart disease and to pay attention to the prognosis of disease symptoms and blood lipid levels. Through syndrome differentiation and treatment, the maximum effect of drug application can be exerted while promoting the improvement of symptoms and blood lipids and reducing the level of cardiac function indicators. It can promote the improvement of myocardial ischemia symptoms, alleviate the damage caused by ischemia and hypoxia to the myocardium, effectively control the body's inflammatory response, provide effective protection to the myocardium, and thereby reduce the levels of various blood lipid indicators. Although the article has achieved good experimental results, some shortcomings remain. The article evaluated the patient's TG and other factors. Still, it did not conduct a correlation analysis on the influencing factors, which cannot fully elucidate the mechanism of action of the combination of BYD-THSWD and Western medicine treatment. Further correlation analysis is needed in subsequent research to elucidate its mechanism of action.

## Conclusion

The clinical relevance of the article lies in the fact that combining BYD-THSWD with Western medicine can improve patients' clinical symptoms. This method can lower the patient's blood lipid levels and various cTnl, NT-proBNF) and MCP-1 indicators. This method can effectively protect the myocardium, improve myocardial ischemia symptoms, alleviate the damage caused by ischemia and hypoxia to the myocardium, and effectively control the body's inflammatory response. In summary, the combination of Western medicine and BYD-THSWD has a good effect on improving clinical symptoms in patients and is worth promoting in clinical application.

## Dodatak

### Ethics approval and consent to participate

The local ethics committee of the Suzhou City Integrated Traditional Chinese and Western Medicine Hospital approved the study. All experiments followed relevant guidelines and regulations, such as the Declaration of Helsinki.

### Consent for publishing

Informed consent was obtained from all subjects and/or their legal guardian(s).

### Availability of data and materials

The original contributions presented in the study are included in the article.

### Funding

Not applicable.

### Author contributions

BW, SZ and RZ participated in the interpretation of results and collection of data, drafting of the manuscript, study concept and design, and study supervision. All authors read and approved the final manuscript.

### Acknowledgements

Not applicable.

### Conflict of interest statement

All the authors declare that they have no conflict of interest in this work.
